# Supplementary material for: nQuire: a statistical framework for ploidy estimation using next generation sequencing
Source: BMC Bioinformatics. 2018 Apr 4;19:122. doi: 10.1186/s12859-018-2128-z (PMC5885312; doi:10.1186/s12859-018-2128-z)
Supplement: Supplementary file 1 — Supplementary figures addressing the effect of denoising, as well as the effect of high coverage outliers on the likelihood of Gaussian and binomial mixtures. (PDF 144 kb) [file 12859_2018_2128_MOESM1_ESM.pdf]

Supplemental Figures

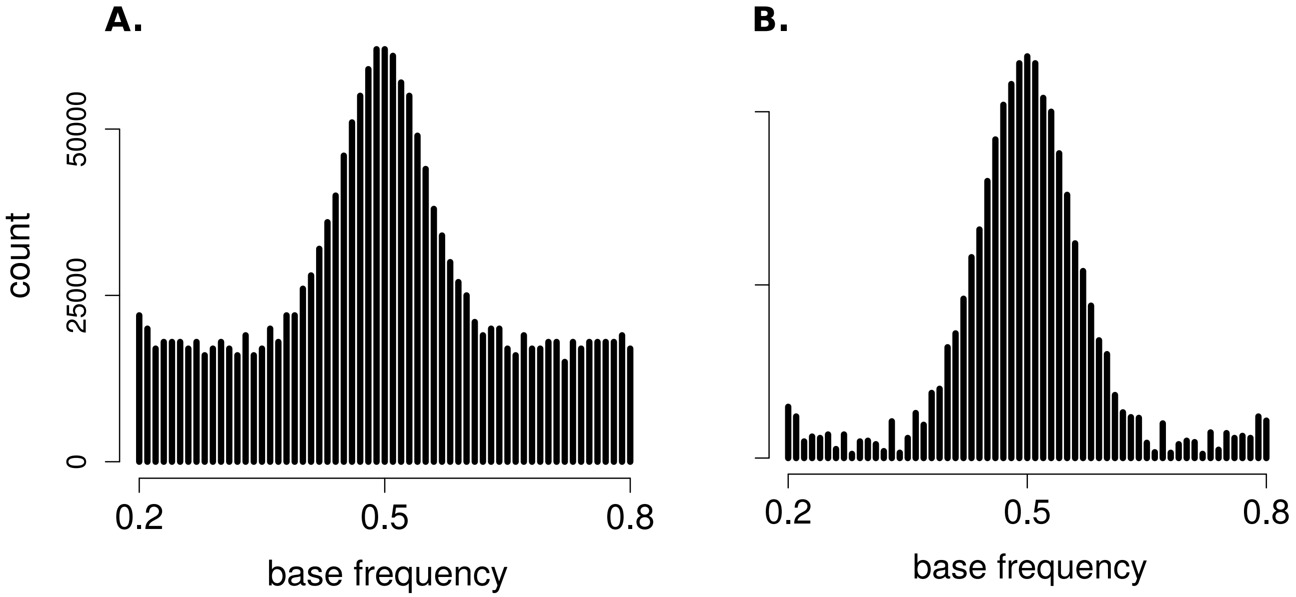

Figure S 1: **Effect of denoising using the uniform mixture component.** (**A.**) The base frequencies of the diploid *P. infestans* sample 99189 before denoising. (**B.**) The base frequencies of the diploid *P. infestans* sample 99189 after denoising using the mixture proportion of the uniform mixture component after likelihood maximization.

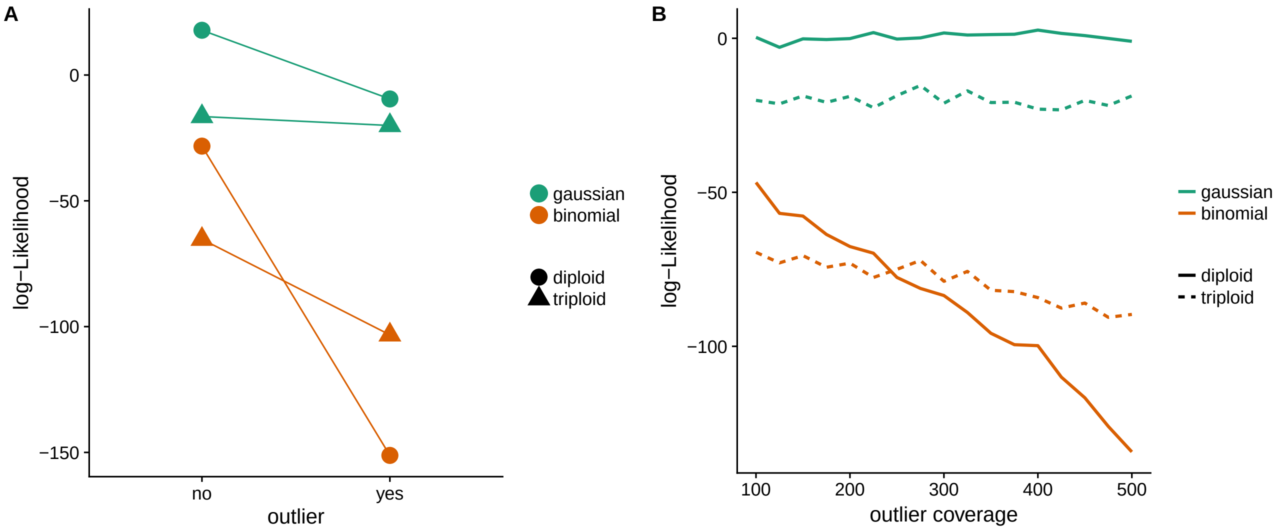

Figure S 2: **Effect of high coverage outliers on the likelihood of Gaussian and binomial mixtures.** The figure shows the results from a simulation of a diploid individual with 10 biallelic sites, sequenced to 100x coverage. (**A.**) We performed the simulation without and with outliers. For the former, we simulated 100x coverage at all 10 positions, and drew a base count from a binomial of size 100 and probability 0.5. For the latter, one out of the ten positions was simulated at 400x coverage and the base count was drawn from the same binomial as above. This simulates a scenario, where reads from three additional (potentially) paralogous, homozygous alleles are falsely aligned to a heterozygous diploid site. In the simulations without an outlier (left column), the diploid fixed model shows a higher log-likelihood than the triploid fixed model both for Gaussian and binomial mixtures. In contrast, in the presence of the high coverage outlier (right column), the drop in log-likelihood for the binomial mixture is more drastic than for the Gaussian mixture, as the high coverage site is given more weight in the binomial. (**B.**) The figure shows how the coverage of the outlier influences the log-likelihood of the binomial mixture. Simulations were performed as in (**A.**) with presence of an outlier, with the difference that the outlier always has a minor allele frequency of 0.2, at different coverages ranging from 100x to 500x. The log-likelihood of the binomial mixture for the diploid fixed model drops below the log-likelihood of the triploid fixed model between 200x and 300x coverage of the outlier.
